# Supplementary material for: Intraspecific variability in plant and soil chemical properties in a common garden plantation of the energy crop Populus
Source: PLoS One. 2024 Oct 21;19(10):e0309321. doi: 10.1371/journal.pone.0309321 (PMC11493264; doi:10.1371/journal.pone.0309321)
Supplement: S1 Fig — PCA analysis of all soil samples (n = 48) collected from variable depths and radial direction of sampling using compositional analysis traits. PC-1 explains 95% of variance is driven primarily by Ca content while PC-2 corresponds to variation in K content. (DOCX) [file pone.0309321.s001.docx]

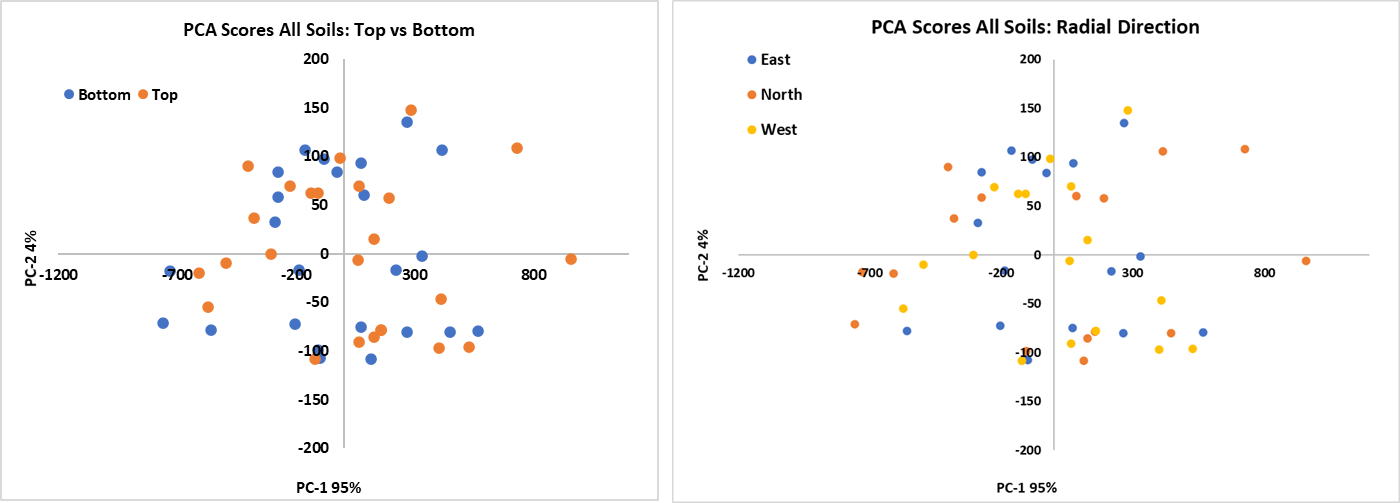


**S1 Fig.**  **PCA analysis**. PCA analysis of all soil samples (n = 48) collected from variable depths and radial direction of sampling using compositional analysis traits. PC-1 explains 95% of variance is driven primarily by Ca content while PC-2 corresponds to variation in K content.
