# Supplementary material for: Intraspecific variability in plant and soil chemical properties in a common garden plantation of the energy crop Populus
Source: PLoS One. 2024 Oct 21;19(10):e0309321. doi: 10.1371/journal.pone.0309321 (PMC11493264; doi:10.1371/journal.pone.0309321)
Supplement: S2 Fig — Boxplots showing pH, nutrient concentrations, and C:N in soils sampled at each of eight focal trees belonging to four different Populus trichocarpa genotypes (delineated with shading). The two replicates of each genotype (BESC 24, BESC 371, BESC 375, BESC 394) are indicated with the suffix A and B along the x-axis. Boxes represent three observations per individual. Units for carbon (C) and nitrogen (N) are percent by mass. Soil pH (pH) and soil C-to-N ratio (CN) are unitless and the remaining nutrients are expressed in mg kg-1. (DOCX) [file pone.0309321.s002.docx]

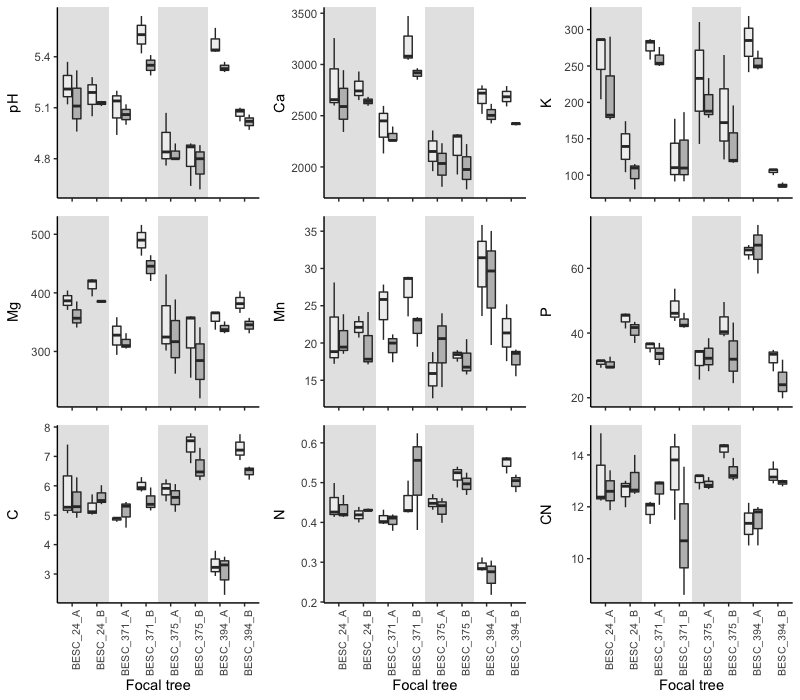


**S2 Fig.** **Soil chemical property variation across individuals and genotypes.** Boxplots showing pH, nutrient concentrations, and C:N in soils sampled at each of eight focal trees belonging to four different *Populus trichocarpa* genotypes (delineated with shading). The two replicates of each genotype (BESC 24, BESC 371, BESC 375, BESC 394) are indicated with the suffix A and B along the x-axis. Boxes represent three observations per individual. Units for carbon (C) and nitrogen (N) are percent by mass. Soil pH (pH) and soil C-to-N ratio (CN) are unitless and the remaining nutrients are expressed in mg kg^-1^.
