# Supplementary material for: Intraspecific variability in plant and soil chemical properties in a common garden plantation of the energy crop Populus
Source: PLoS One. 2024 Oct 21;19(10):e0309321. doi: 10.1371/journal.pone.0309321 (PMC11493264; doi:10.1371/journal.pone.0309321)
Supplement: S3 Fig — PCA analysis of chemical properties of all soil samples collected from 0–10 cm depth for BESC 24 (low lignin) and BESC 375 (high lignin). Twelve observations for each genotype. (DOCX) [file pone.0309321.s003.docx]

**S3 Fig. Targeted PCA analysis.** PCA analysis of chemical properties of all soil samples collected from 0-10 cm depth for BESC 24 (low lignin) and BESC 375 (high lignin). Twelve observations for each genotype.
