# Supplementary material for: Intraspecific variability in plant and soil chemical properties in a common garden plantation of the energy crop Populus
Source: PLoS One. 2024 Oct 21;19(10):e0309321. doi: 10.1371/journal.pone.0309321 (PMC11493264; doi:10.1371/journal.pone.0309321)
Supplement: S4 Fig — Bulk density data corresponding to soil cores obtained from (Blue) “Top” soil cores [soil cored at the 0”-2” depth from surface of the soil horizon] and (Green) “Bottom” soil cores [soil cored at the 2”-4” depth from surface of the soil horizon]. Four observations per genotype included two observations each for “top” or “bottom” cores. (DOCX) [file pone.0309321.s004.docx]

**S4 Fig. Bulk density data.** Bulk density data corresponding to soil cores obtained from (Blue) “Top” soil cores [soil cored at the 0”-2” depth from surface of the soil horizon] and (Green) “Bottom” soil cores [soil cored at the 2”-4” depth from surface of the soil horizon]. Four observations per genotype included two observations each for “top” or “bottom” cores.
