# Supplementary material for: Intraspecific variability in plant and soil chemical properties in a common garden plantation of the energy crop Populus
Source: PLoS One. 2024 Oct 21;19(10):e0309321. doi: 10.1371/journal.pone.0309321 (PMC11493264; doi:10.1371/journal.pone.0309321)
Supplement: S1 Table — Variance in soil biogeochemical properties explained (Partial R2) by linear models containing either individual trees (n = 3 soil samples per tree) or genotypes (2 trees per genotype, n = 6). Individual trees were significantly related to soil properties (p < 0.001) for all measured variables. No statement about statistical significance is made about genotype effects due to insufficient replication at this level of analysis. A significant effect of sample depth is denoted with an asterisk (*) for response variable. (DOCX) [file pone.0309321.s005.docx]

**S1 Table. Variance in soil biogeochemical properties.** Variance in soil biogeochemical properties explained (Partial R^2^) by linear models containing either individual trees (n = 3 soil samples per tree) or genotypes (2 trees per genotype, n = 6). Individual trees were significantly related to soil properties (*p* < 0.001) for all measured variables. No statement about statistical significance is made about genotype effects due to insufficient replication at this level of analysis. A significant effect of sample depth is denoted with an asterisk (*) for response variable.

|  | Partial *R^2^* | |
| --- | --- | --- |
| Variable | Individual | Genotype |
| pH* | 0.85 | 0.54 |
| Ca* | 0.79 | 0.53 |
| K* | 0.76 | 0.01 |
| Mg* | 0.72 | 0.27 |
| Mn | 0.51 | 0.26 |
| P* | 0.87 | 0.15 |
| C | 0.86 | 0.19 |
| N | 0.76 | 0.01 |
| C:N | 0.35 | 0.18 |
