# Supplementary material for: Intraspecific variability in plant and soil chemical properties in a common garden plantation of the energy crop Populus
Source: PLoS One. 2024 Oct 21;19(10):e0309321. doi: 10.1371/journal.pone.0309321 (PMC11493264; doi:10.1371/journal.pone.0309321)
Supplement: Attachment — Submitted filename: Review report PLONE.docx [file pone.0309321.s007.docx]

**Intraspecific variability in plant and soil chemical properties in a common garden plantation of the energy crop Populus**

The study on the effects of *Populus* genotypes on physiochemical properties of soil, C accumulation and elemental content in shoots and roots is interesting. The concept of the study to reduce atmospheric carbon dioxide content through candidate plant genotype that promotes soil C accretion and plant phenotype is important in climate change context. The manuscript is well drafted, results are well explained. However, there are some corrections that the authors have to go through it.

1. Line 38 Specify plant tissues as roots and stem.
2. Line 41-42 Modify the line “the potential for co-optimizing above-belowground plant systems”.
3. Line 46 The above and below ground properties have not measured in the study. The authors have only measured nutrient composition so instead of “property” the nutrients/chemical components/elements” words are more appropriate.
4. Line 107 The study is not focused on the microbial traits/diversity, so I think donot mention it here because the paragraph is mainly directing the hypothesis and objectives of the study.
5. Line 117 -122 The hypothesis of the study should be mentioned first followed by the objectives. Moreover, the hypothesis should be in sentence form rather than in points.
6. Line 120-122 is the part of objective not hypothesis so rearrange it.
7. Line 107-122 Needs to be rewritten based on the above comments.
8. Line 128-132 Did the authors collect the soil samples from the plain land which was earlier used for populus plantation. If this is the case the land surface should be uniform throughout then what the authors are meant by under the planted and non-planted areas. And if the two areas differ in terms of plantation, then I am wondering how the soil properties should be same.
9. With the under-plant samples did the author means rhizosphere samples.
10. Line 138 How these soil samples differ from the samples mentioned in the above paragraph (line 128-132)
11. Line 140 Two replicates are very few, atleast five replicates should be taken for the good results.
12. Line 145 For tree breast height. Mention the exact height from the surface also.
13. Line 146. Mention the depth from the surface at which the root samples were collected.
14. Line 203 The text did not match Fig. 2A. According to the figure the Ca content is higher at 5-10 cm in all the genotypes not on the soil surface. Check it.
15. Line 220 -221 It is better if the increasing results of Ca, Mg and K in BESC 24 is shown in the % increase compared to other genotypes.
16. Line 229 Replace properties with chemical traits see the above comment 3.
17. Above-ground not only includes stem but leaves too then why the authors have not measured the chemical constituents in leaves.
18. Line 242 see the above comment on property.
19. Insert figure/table number in the discussion section while discussing your results so that it is easy for the readers to understand.
20. Line 295 see the above comment on property
