# Supplementary material for: Intraspecific variability in plant and soil chemical properties in a common garden plantation of the energy crop Populus
Source: PLoS One. 2024 Oct 21;19(10):e0309321. doi: 10.1371/journal.pone.0309321 (PMC11493264; doi:10.1371/journal.pone.0309321)
Supplement: Attachment — Submitted filename: Response to Reviewers.docx [file pone.0309321.s008.docx]

Dear Dr. Albrectsen,

Thank you for your letter of 28 May 2024 regarding our manuscript “Intraspecific variability in plant and soil chemical properties in a common garden plantation of the energy crop *Populus*”. We appreciate the positive response to our paper and the constructive and thoughtful comments from you and from the peer reviewers. We have carefully addressed each comment and now present a revised manuscript.

Below please find review comments in bold, followed by our point-by-point response to each reviewer comment. Revisions made to the main document are in red font, and line numbers in our responses refer to those in our revised manuscript. We have additionally made changes to format the manuscript in accordance with PLOS ONE guidelines. Please do not hesitate to contact me if you have any questions.

Sincerely,

Udaya Kalluri (on behalf of all co-authors)

**Editor comment to authors:**

**Thank you for submitting your manuscript to PLOS ONE. After careful consideration, we feel that it meets PLOS ONE’s publication criteria after miner revisions. Therefore, we invite you to submit a revised version of the manuscript that addresses the points raised during the review process.please go through figures and make sure the the colors can be distingushed. The choice of dark purple is difficult to tell from the dark blue in the second figure.**

Thank you for this feedback. We have reviewed all figures and modified Fig 2 to ensure the colors can be distinguished. The new figure presents panel 2B as a side-by-side bar plot, with the dark purpled changed to red.

**I also suggest that sample sizes are indicated in the legends throughout.**

Thank you. We have added samples sizes to each table and figure legend in the manuscript.

**Reviewer #1: Very interesting research in dependence (connection) and mutual influence between soil and plant through quantitative analysis of micro and macro elements. The authors set up the experiments well, emphasizing individual and genotypic differences in one species of poplar (Populus trichocarpa). Numerous collected results mainly prove individual variability (therefore also genotypic) significant dependence for most of the observed properties between soil and plant, but also a weak correlation for properties of the above-ground and underground parts of the tree.**

Thank you for your thoughtful review of our manuscript.

**I recommend that the paper be published with a few questions for the authors to think about or possibly supplement the text in the paper:
1) Is the observed interdependence of properties a result of the plant's influence on the soil or vice versa?**

This is a good point. However, while we did observe some relationships between plant and soil traits (e.g. soil N relations with plant K), we actually conclude that there is not consistent evidence for such interdependence (lines 303-305). Where we did observe differences was among individuals and potentially genotypes. Here, we assume that differences among individuals are mostly result of the plant, because these trees were planted on a common soil, with locations randomized within spatial blocks. We nonetheless acknowledge that soils are inherently heterogeneous and this heterogeneity could result in variation in plant traits (lines 269-271).

**2) Are there any available data regarding soil properties before the planting of these genotypes, or were they exclusively taken from the control plot?**

Because this plot was established to investigate variation in plant—and not soil—traits, there are no data or archived soil samples available which predate the establishment of the plantation. Thus, our analysis focuses on differences among individuals and genotypes. We assume that these differences represent deviation from a common historical baseline, but because these comparisons are an imperfect characterization of these trajectories, we have avoided discussing rates of change over time.

**3) Should all of this be verified through controlled (closed) experiments, such as in a greenhouse using potted substrate?**

An excellent suggestion. Our study (pilot) was a first look into whether plant-soil relationships exist, and if so, whether such plant-soil relationships can be meaningfully tracked as a factor of plant genotype and soil nutrient level in an intermixed plantation. While follow-up experiments in controlled (closed) environment to further evaluate the strong relationships observed in the field will be insightful mechanistically, this is beyond the scope of the present manuscript—in which we focus on the applicability of long-term genetic diversity plantations. As a side note, a new controlled greenhouse plant x soil study is currently underway focused on comparing reciprocal effects of soil types (potting mix/field soil) and chemically divergent plant genotypes.

**Reviewer #2: Intraspecific variability in plant and soil chemical properties in a common garden plantation of the energy crop Populus
The study on the effects of Populus genotypes on physiochemical properties of soil, C accumulation and elemental content in shoots and roots is interesting. The concept of the study to reduce atmospheric carbon dioxide content through candidate plant genotype that promotes soil C accretion and plant phenotype is important in climate change context. The manuscript is well drafted, results are well explained. However, there are some corrections that the authors have to go through it.**

Thank your review and your thorough comments.

**1. Line 38 Specify plant tissues as roots and stem.**

We have made this change (lines 34-35).

*Lines 34-35: Here, we provide initial observations of C and nutrients in soil and root and stem tissues from a common garden…*

**2. Line 41-42 Modify the line “the potential for co-optimizing above-belowground plant systems”.**

We have edited this line for clarity (lines 37-39)

*Lines 37-39: Our goal was to explore the value of such field sites for evaluating genotype-specific effects on soil C, which ultimately informs the potential for optimizing bioenergy systems for both aboveground productivity and belowground C storage.*

**3. Line 46 The above and below ground properties have not measured in the study. The authors have only measured nutrient composition so instead of “property” the nutrients/chemical components/elements” words are more appropriate.**

Thank you. We have replaced the generic word “property” to more specific reference to “soil chemical properties/chemistry/chemical trait/elements/nutrient” throughout the manuscript as suggested by the reviewer (line 42-43). We the term “property” is retained only where general reference to soil characteristics is implied.

*Line 42-43: While correlations among elements were observed both within and among sample types (soil, stem, root), above-belowground correlations were generally poor.*

**4. Line 107 The study is not focused on the microbial traits/diversity, so I think donot mention it here because the paragraph is mainly directing the hypothesis and objectives of the study.**

We have made this change (line 103).

*Lines 103: Replication of genotypes in blocks provides the opportunity for capturing genotypic effects, but belowground soil and plant traits have not yet been studied in this population.*

**5. Line 117 -122 The hypothesis of the study should be mentioned first followed by the objectives. Moreover, the hypothesis should be in sentence form rather than in points.**

Thank you for this suggestion. We agree and have moved the hypotheses to precede the objectives of the study. Additionally, the hypotheses are now stated in three separate sentences (lines 102-119).

*Lines 100-119: In the present study, we leveraged a genetically diverse, long-term P. trichocarpa population—with a wide range of phenotypic variation reported in aboveground traits—growing in a common garden field setting in the Pacific Northwest, USA (20). Replication of genotypes in blocks provides the opportunity for capturing genotypic effects, but belowground soil and plant traits have not yet been studied in this population. We hypothesized that distinct P. trichocarpa genotypes determine variation in soil properties, and that this variation is observable at the scale of individual trees. We also hypothesized that plant lignin phenotype influences amount of C in soil and that belowground traits correlate positively with aboveground traits across genotypes. We evaluated these hypotheses using a woody perennial bioenergy plant type in a ten-year old intraspecific GWAS site. Our objectives were to evaluate 1) the potential of focal tree soil sampling for detecting associations of poplar genotypes with soil biogeochemistry (e.g. C, nitrogen (N), phosphorus (P), and a range of soil micro- and macronutrient elements) and soil bulk density, and 2) the strength of correlations between above- and belowground traits (stem vs root and plant vs soil). To do this, we measured soil, root, and stem chemical traits for several focal trees spanning four different P. trichocarpa genotypes representative of population extremes in cell wall chemistry (Fig 1A)—i.e. phenotypes with low (BESC 24) or high (BESC 375, BESC 371, BESC 394) lignin content based on population-wide cell wall chemistry data previously reported for this population (13,20–23). We also explored the influence of sampling location on measured soil properties, potential effects of plant tissue chemistry on soil C, relationships among nutrient concentrations among plant and soil pools.*

**6. Line 120-122 is the part of objective not hypothesis so rearrange it.**

See response to #5. This sentence is now included along with the study objectives (lines 117-119).

**7. Line 107-122 Needs to be rewritten based on the above comments.**

See response to #5. This section has been re-written as described by the reviewer (lines 100-119)

**8. Line 128-132 Did the authors collect the soil samples from the plain land which was earlier used for populus plantation. If this is the case the land surface should be uniform throughout then what the authors are meant by under the planted and non-planted areas. And if the two areas differ in terms of plantation, then I am wondering how the soil properties should be same.**

Yes, unplanted areas were previously under the same *Populus* plantation management. They were simply unplanted in the current experimental design. We have clarified this in the text (lines 135-137).

*Lines 135-137: For this study, we collected surface soil (top 10 cm) from planted areas and from areas that do not contain Populus individuals in the current plantation.*

**9. With the under-plant samples did the author means rhizosphere samples.**

We did not make any attempt to separate the rhizosphere soils from the bulk soils as we were primarily interested in the effects of trees on overall soil chemical traits. We have added the word “bulk” to avoid any confusion (line 145).

*Line 145: Bulk soil samples (0-5 cm [top] and 5-10 cm [bottom] depth) were collected…*

**10. Line 138 How these soil samples differ from the samples mentioned in the above paragraph (line 128-132)**

These are the same samples and we have added text to make this clear (line 137). In the previous paragraph, we present averages to give context for mean soil characteristics, while the results focus on differences among samples, individuals, and genotypes.

*Line 137: Analysis of basic soil characteristics at the time of sampling revealed an average pH…*

**11. Line 140 Two replicates are very few, at least five replicates should be taken for the good results.**

We agree that this is low replication for a comparison among genotypes. This effort focused on characterizing variation at the scale of individuals. We do make comparisons among genotypes, but included text to emphasize that these comparisons should be interpreted with caution given the low replication (e.g. lines 181-183). We have added an additional note of caution to the discussion (lines 298-301).

*Lines 181-183: To provide an initial look at whether soil variables are clustered with respect to genotype, we additionally calculated partial R^2^ for models fit with genotype and sample depth as predictors. However, these analyses are considered exploratory due to inadequate replication.*

*Lines 298-301: Most importantly though, our findings of differences among focal trees and trends toward genotype differences pave the way for a more comprehensive and well-replicated comparison of genotype-specific effects in this system.*

**12. Line 145 For tree breast height. Mention the exact height from the surface also.**

We used the standard ~1.3 m and have added this information to the text (line 152).

*Line 152: Stem cores (1 cm in diameter) from tree breast height (~1.3 m) were collected…*

**13. Line 146. Mention the depth from the surface at which the root samples were collected.**

We have added a sentence to describe the depth from surface of root samples (lines 155-156).

*Lines 155-156: We targeted the surface-most roots which usually corresponded to a depth of less than 10 cm from the surface.*

**14. Line 203 The text did not match Fig. 2A. According to the figure the Ca content is higher at 5-10 cm in all the genotypes not on the soil surface. Check it.**

This is an excellent catch. Thank you. The text is correct, but the legend was wrong for Fig 2. We have amended the figure such that the green boxes are from the 0-5 cm samples and the blue boxes are from the 5-10 cm samples. Additionally, we have rearranged the figure so that the 0-5 increment is on the left and the 5-15 increment is on the right. See Reordered bar graphs below:

**15. Line 220 -221 It is better if the increasing results of Ca, Mg and K in BESC 24 is shown in the % increase compared to other genotypes.**

We prefer to be able to show the variation among the other genotypes. Nonetheless, we think the reviewer might find the stacked bars difficult to interpret. Thus, we have changed panel 2B to separate the bars of the bar plot for easier comparison among genotypes.

**16. Line 229 Replace properties with chemical traits see the above comment 3.**

We have replaced this term with “chemical traits” (line 250-251).

*Line 250-251: Extent of correlation among aboveground-belowground chemical traits*

**17. Above-ground not only includes stem but leaves too then why the authors have not measured the chemical constituents in leaves.**

For the purpose of evaluating and estimating bioenergy feedstock productivity and composition, the research community refers simply to productivity and composition of usable lignocellulosic biomass, stem (biofuel feedstock). Therefore, the design of our pilot study included a root and soil property correlation with a previously well-characterized aboveground reference, *Populus* stem. Given the goals of our study, our pilot study did not include logistically challenging, genotype-specific collection of leaf material from the tall mature trees (height ranging from 20-50 m).

**18. Line 242 see the above comment on property.**

We have replaced this term with “chemical traits” (line 264).

*Line 264: Insights from our pilot study represent a first report on aboveground-belowground chemical trait correlations for different P. trichocarpa genotypes…*

**19. Insert figure/table number in the discussion section while discussing your results so that it is easy for the readers to understand.**

We have selectively inserted references to particularly relevant figures and tables throughout the discussion text.

**20. Line 295 see the above comment on property**

We have replaced this term with “chemical traits” (321).

*Line 321: Though select correlations between plant and soil properties were detected, we generally observed poor correlations among above- and belowground chemical traits.*
